# Effects of access to a well-resourced environment on dairy calf cognition and affective state

**DOI:** 10.1371/journal.pone.0323089

**Published:** 2025-05-16

**Authors:** Malina Suchon, Daniel M. Weary, Marina A. G. von Keyserlingk

**Affiliations:** Animal Welfare Program, Faculty of Land and Food Systems, The University of British Columbia, Vancouver, British Columbia, Canada; Michigan State University, UNITED STATES OF AMERICA

## Abstract

Dairy calves are often raised without maternal contact and in environments of low complexity. Environments that limit natural behaviors are known to impair cognitive development and affective states. We explored the effect of environmental complexity on one measure of social cognition (the ability to discriminate between conspecifics) and one measure of affective states (sensitivity to reward). Pairs of calves were randomly allocated to either 1) pair housing for 22.5 h/d with 1.5 h of daily access to a well-resourced pen which included 3 other calves and physical devices (Enriched; *n* = 6 pairs) or, 2) pair housing for 24 h/d (Control; *n* = 6 pairs). Calves were trained to discriminate between 2 calves in a Y-maze. Twelve of the 24 calves tested met the learning criterion, requiring 15.7 ± 2.59 (mean ± SD) training sessions. Treatment did not affect the number of sessions needed to reach the learning criterion. Calves were then subjected to a Successive Negative Contrast test during which they were trained to approach a 0.5 L milk reward over 3 trials/day for 3 days. On the last training day, latencies of enriched calves increased over daily trials while latencies for control calves were lower and remained relatively consistent, indicative of greater sensitivity to reward. Starting on day 4, the reward was reduced to 0.1L of milk/trial and remained at this level for the next 5 test days. Latency to reach the reward increased across trials within each test day, but no effect of treatment or test day was found. Our findings suggest that calves can discriminate among individuals but learning was not affected by treatments. Calves raised in standard pair housing showed increased sensitivity to reward, consistent with experiencing a more negative emotional state in comparison to calves reared with access to a well-resourced environment.

## Introduction

Under naturalistic conditions, calves are able to engage socially with conspecifics of diverse ages and navigate an environment that varies in biotic and abiotic factors [1]. In contrast, on many dairy farms calves are separated from their dams at birth and raised during the milk feeding period individually in pens with little physical complexity and no social contact [2]. While some farms raise their milk-fed calves in social groups, these environments still typically provide little to no complexity otherwise [2–4]. Environments with low complexity (both social and physical components) limit opportunities for calves to express natural behaviors [5], such as foraging [6], or social interactions with peers [7], and can impair cognition and welfare [6,8,9].

How the complexity of the rearing environment affects cognitive development has received considerable interest in the research community. For instance, improvements in spatial learning have been observed with the addition of species-relevant physical items (fish: [10]; rats: [11]; pigs: [12]), and more space (hens: [13]). Social isolation can impair social recognition memory in rats [14], but this effect can be reversed by providing odor-enriched housing [15]. In cattle, the complexity of the social group improves behavioral flexibility (maternal contact, group and pair housing: [16]; pair housing: [17]). Calf cognition is also affected by nutritional complexity; calves provided grain and hay showed improved performance in reversal learning compared to calves provided only grain early in life [18,19]. Zhang et al. [20] reported that calves raised in pens containing a number of physical items (i.e., hay net, rubber teat, plastic chain, stationary brush) tended to show improved object recognition compared to calves housed in barren pens.

Environmental complexity can impact affective state [9]. Play behavior is reduced when calves have more limited space allowance [21,22], commonly associated with individual housing [23,24]. According to Bučková et al. [25], when subjected to a spatial judgment bias task, pair-housed calves responded more positively to ambiguous cues than did individually housed calves, a response bias indicative of positive affective states. More broadly, research has found evidence of more optimistic judgment bias in animals housed in more complex environments for pigs [26], sheep [27], horses [28], and European starlings [29].

Although a growing body of work has investigated the effects of additional resources to calf housing, knowledge about effects on cognition and affective states in dairy calves is still scarce. Individual recognition (i.e., the ability to identify another organism according to its unique characteristics [30]) can be inferred from cattle’s relatively stable hierarchy [31]. Considered a pre-requisite of individual recognition, the ability to discriminate among conspecifics has been reported in adult dairy cows [32–34] but little is known about this ability at younger ages. Y-maze testing has been used to investigate the ability of cattle to discriminate between conspecifics (using live stimuli: [33]; pictures of conspecifics [32,34]) as well as other stimuli (e.g., [17,35]). As described by Gheusi et al. [36], this method is commonly used to train animals to respond differentially to the presentation of two conspecifics. In brief, subjects are reinforced when entering the arm containing the positive stimulus, and the proportion of rewarded responses is measured across sessions. The task is considered learned when the subject performs above a pre-establish learning criterion, based on the probability of this performance occurring by chance (e.g., [33,34,37]).

Affective states, i.e., emotions and other feelings experienced as pleasant or unpleasant [38], can be assessed using sensitivity to reward loss; animals experiencing negative affective states are expected to show enhanced responses to a reduction in reward [39]. For example, Burman et al. [40] reported that rats housed in barren environments were slower to reach a food reward available in a test arena after the reward had been unexpectedly reduced and that this response lasted longer compared to rats housed in enriched environment. Similarly after being trained to reach a reward consisting of six pieces of fruit (i.e., 6 apples or 6 bananas), pigs raised in barren pens took longer to access the reward when it was reduced to a single piece of fruit, compared to the pigs reared in enriched pens [41]. Neave et al. [42] compared pair-housed calves, reared in either basic or well-resourced pens, and provided with access to an additional pen for 30 min/d. When the complexity of the reward pen was unexpectedly reduced, barren-housed calves showed a decrease in anticipatory behaviors (i.e., fewer behavioral transitions), suggesting that these calves were more sensitive to the reward reduction compared to calves reared in well-resourced environments. This latter study also suggested that even temporary access to a complex environment can be valuable, as it has been shown for mice [43] and rats [44,45]. Little work to date has investigated the benefits of temporary access to a well-resourced pen on dairy calf cognition and affective state.

The aim of this study was to investigate whether providing dairy calves temporary access to a more complex environment affects their ability to discriminate between conspecifics and their sensitivity to reward loss. We predicted that calves with daily access to a well-resourced pen would be faster to reach the learning criterion in the social discrimination task, and show a higher learning rate compared to control calves reared without access to the well-resourced pen. We also hypothesized that calves raised in control conditions would respond more strongly to reward loss, as evidenced by increased latencies to access the reward.

## Materials and methods

This study was conducted at The University of British Columbia’s Dairy Education and Research Center (Agassiz, British Columbia, Canada) between August 2023 and March 2024. All procedures used were approved by the UBC Animal Ethics Committee under protocol #A23-0090. Sample size was based on previous studies investigating the effect of housing on dairy calves’ cognition (8–10 individuals per treatment group, [16–18]) and works assessing animals’ affective states using a Successive Negative Contrast paradigm (6–16 individuals per treatment group [40,41,46]). Given this range and our technical limitations, we decided on a sample size of 12 individuals per treatment.

### Animals and housing

In this study, we used 24 Holstein heifers designated as focal calves. All focal calves (birthweight: 37.9 ± 3.9 kg; mean ± SD) were permanently separated from their dam within 12 h after birth, fed 4 L of > 50 g/L of IgG colostrum, and then kept in individual pens for the first 5 d of life. Calves were then enrolled in blocks of 2 pairs of individuals born within 4 d of each other with birthweight differences no larger than 10 kg. Each pair of calves was assigned one of the treatments: (1) pair housing for 22.5 h/d and 1.5 h of daily access to a well-resourced pen (Enriched; 6 pairs of calves) or (2) pair housing for 24 h/d (Control; 6 pairs of calves). Treatments were allocated at random within blocks using a table and a random number generator (using the Excel function *RAND*), ensuring that one pair of each treatment was always tested simultaneously. As subsequent blocks were enrolled, we monitored pairs’ birthweight (control: 37.8 ± 3.5 kg; enriched: 38.2 ± 4.6 kg) and age differences (control: 1.5 ± 1.0 d; enriched; 1.0 ± 0.9 d) to balance these across treatments.

**Fig 1:**
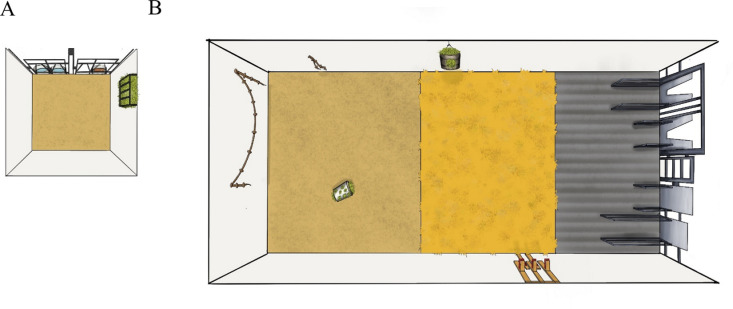
(A) Home and (B) enriched pens. All focal calves (n = 24) were pair-housed in pens bedded with sawdust (A; in 2.4 m x 2.0 m). Pairs of calves assigned to the enriched treatment (n = 12) were provided daily access for 1.5 h to a well-resourced pen (B, 11.8 m x 4.8 m). This pen was covered by three different types of flooring: the first third of the pen was concrete flooring covered with sawdust, the middle third was covered with straw, and the remaining third was plastic covered slatted flooring. The pen also contained vertical and horizontal ropes, a pierced barrel filled with hay, a hay bag, a stationary brush, and three other calves.

At 6 d of age, pairs of calves (n = 12) were moved into pair housing (Fig 1A) where they remained for the duration of the study. Pens measured 2.4 m x 2.0 m, were bedded with sawdust, and included *ad libitum* access to starter grain and water in buckets, and hay in a rack. Calves received 8L/d of whole milk from a bottle divided into 2 meals of 4 L (0800 and 1600). These indoor pens were in a purpose-built and naturally ventilated calf barn with natural light following seasonal variation supplemented with continuous dimmed artificial lights.

The enriched calves had daily access to a well-resourced pen (Fig 1B) providing more space, physical devices and social partners than their home pens. The well-resourced pen measured 11.8 m x 4.8 m with the floor space divided into 3 sections: the first third was filled with sawdust, the middle third with straw, and the last third covered with plastic covered slatted flooring. Integrated into the pen were several additional items, including: 1) vertical and horizontal ropes, 2) a pierced barrel filled with hay, 3) a hay bag, and 4) a stationary brush station. Each pair of calves provided access to the well-resourced pen (n = 6) was also mixed with a set of 3 companion calves (6 sets; age difference between the focal calves and the companion calves averaged 10.7 ± 5.0 d). Each set of companion calves was formed with 3 Holstein heifers. With the exception of when the calves were moved into the well-resourced pen (timing described below), the companion calves were housed together in groups of 10 calves.

Once per day over 7 weeks, calves were gently led by handlers (i.e., 4 different trained handlers alternating across days) from their home pen to the well-resourced pen, starting with the companion calves and then followed by the enriched calves. After 90 minutes of access, all calves were brought back to their home pens. The well-resourced pen was located in the same barn as the calves’ home pens, with the closest pen located 35 m and the farthest pen 45m away; access the well-resourced pen occurred between 11:00 and 15:00 h.

The decision to include the companion animals in sets of three was based on having treatments that contrasted in both physical and social components. The duration of the access was determined following the observation of a pilot group where we noted that calves were most active within the first 60 minutes of access to the well-resourced pen, and most calves lying down within 90 min. Thus the 90 min period was chosen to allow sufficient time for calves to be active and to rest.

### Discrimination task

**Fig 2:**
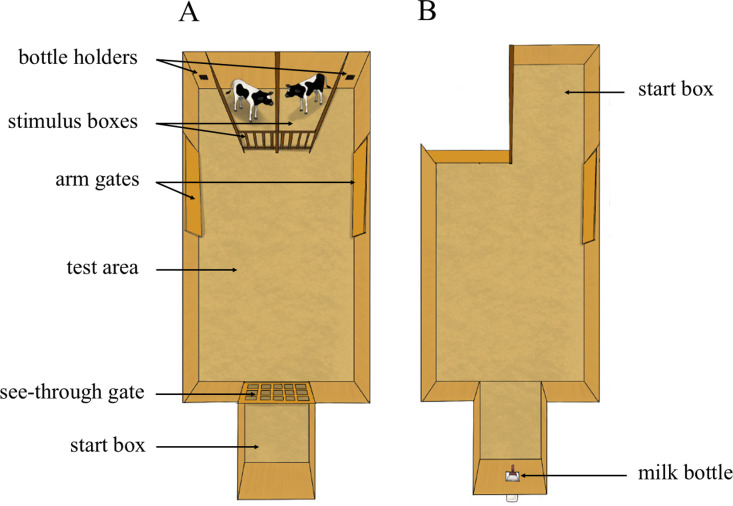
Discrimination task arena (A) and the Successive Negative Contrast test arena (B). Calves were trained to discriminate conspecifics in a Y-maze apparatus (A) consisting of a 2-compartments stimulus box (2.4 m x 3.4 m), a test area (5 m x 3.3 m), and a start box (1.8 m x 1.3 m), all bedded with sawdust. Two gates prevented access from the start box to the test area: an opaque gate and a see-through gate that provided a view of the test area and the stimuli boxes. Each arm of the Y-maze contained a bottle holder, and a gate capable of limiting view and access to the arm. During training and test sessions, calves were rewarded when entering the arm on the S+ side by placing a bottle of milk in the bottle holder and allowing access 0.2L of milk. The apparatus was modified for the Successive Negative Contrast tests (B): the stimulus box was switched with a 1.6 m x 2 m start box; opaque and see-through gates were removed creating a 1.8 m x 1.3 m alley where a milk bottle was hung at the midpoint along the back wall. See S1 Fig in supplementary materials for tests timeline.

Focal calves (n = 24) were trained by 4 experimenters working in pairs, to discriminate between conspecifics in a Y-maze apparatus (Fig 2A). Each focal calf was assigned two unfamiliar stimulus calves and managed such that one pair of control calves and one pair of enriched calves shared the same stimuli calves. Stimulus calves (n = 6 sets) were Holstein heifers of similar age (2.4 ± 1.3 d of age difference) and birthweight (6.4 ± 5.8 kg). The focal calf was always exposed to the same stimulus calves throughout the duration of the discrimination task. One of the 2 stimulus calves was randomly assigned as positive (S+) and the other as negative (S-). During testing, stimulus calves were placed in the stimulus boxes. Stimulus calf location varied within test sessions following the constraints that: 1) each stimulus calf appeared an equal number of times on each side, and 2) each stimulus calf appeared on the same side for no more than 2 consecutive trials. Training for the discrimination task consisted of 3 training sessions and 17 testing sessions, all occurring between 8:00 and 10:30 over 20 consecutive days. On the morning of each session, the milk ration was withheld until testing began. Calves were provided their morning allotment of milk over the session and any remaining milk from the 4L-morning feeding was delivered to the calves once they returned to the home pen.

#### Training sessions.

Before training, calves were habituated to the test arena over 2 days. On the first day, calves spent 10 min in the empty arena. The following day, the S+ calf was placed in the stimulus box and the focal calves were exposed for another 10 min. After 10 d of treatment exposure (i.e., access to the well-resourced pen for enriched calves, and regular pair housing for control calves), when calves were 17.3 ± 1.2 d old (control: 17.6 ± 1.2 d; enriched: 17.0 ± 1.3 d), they were subjected to training sessions over 3 consecutive days. Each training session included 10 trials performed with only the S+ calf placed in the stimulus boxes. The 2 first training sessions began with ‘guided trials’ (d1: 4 first trials, d2: 2 first trials), meaning that an experimenter guided the focal calf to the S + arm using gentle hand contact, which were then followed by regular trials. For each trial, the focal calf was gently moved to the start box with both gates (i.e., opaque and see-through gates) to the test arena closed. First, the opaque gate was opened to allow the calf to view the stimulus boxes through the see-through gate. After 10s, the second gate was removed releasing the calf into the test area. The calf was given 1 min to enter the S+ side which was rewarded with approximately 0.2L of milk via a nipple bottle (i.e., calves drank for up to 8 seconds). The trial ended when the calf had consumed all the milk; the calf was then brought back to the start box where she had access to milk in a bottle for 3 seconds. This procedure was repeated until a total of 10 trials were completed. If the calf took more than 1 min to enter the rewarded side, an experimenter gently intervened and guided the calf to the S+ side.

During the 3^rd^ training session (i.e., 3^rd^ day of training), 2 trials were performed with the S- calf instead of the S+ calf. For these trials, entering the S- side was punished with a 1-min time-out in the test area. During a time-out, a solid gate prevented the focal calf from having visual contact with the other side, and the start box gates were closed. Once the time-out ended, the calf was brought back to the start box for the next trial.

#### Testing sessions.

From the 4^th^ to the 20^th^ day of the discrimination task, calves were trained with exposure to both S + and S- calves simultaneously. The first testing session began with 4 ‘guided trials’, followed by 6 regular trials. Every subsequent testing session consisted of 10 regular trials. During each trial, focal calves were released in the arena as described for the training phase, but with both stimulus calves present; focal calves were allowed the opportunity to approach one stimulus calf per trial. Entering the S + arm was rewarded (with 0.2L of milk) and entering the S- side was punished with a 1-min time-out in the test area.

Over the testing phase, 6 calves (3 enriched calves and 3 control calves) performed one shorter test session (i.e., less than 10 trials) owing to a lack of motivation to drink. Ten calves were provided breaks between 2 test days for health reasons: 4 of these calves were diagnosed with a respiratory infection and were given an injection of antibiotic (*Resflor,* Intervet Inc.); one stimuli calf was diagnosed with a respiratory infection resulting in a 1-d break for 2 focal calves; and finally, technical difficulties resulted in 2-d breaks between test sessions for 4 calves (age: 24 ± 1.1 d).

### Successive negative contrast

Sensitivity to reward loss was assessed using a Successive Negative Contrast test (Flaherty, 1996). In brief, calves were trained to drink a 0.5 L milk reward in an arena 3 times per day over 3 days. From the 4^th^ to 8^th^ day, the milk reward was reduced to 0.1 L. The apparatus consisted of a start box, a rectangular area, and an alley where a milk bottle was hung at the midpoint along the back wall (Fig 2B).

#### Training.

Daily training sessions started at 36 d of treatment exposure (i.e., 44.2 ± 1.3 days of age, control: 43.6 ± 1.4 d; enriched: 42.8 ± 1.2 d) and were performed between 8:00 and 9:00. The morning milk ration was withheld to increase motivation for the milk reward during training. The training session began with the calf placed into the start box for 1 min before the door was opened allowing access to a milk bottle with 0.5L of whole milk. The trial ended when the calf had consumed all of the milk; each daily training session consisted of 3 trials, collectively providing 1.5 L of the daily milk ration. On the first day, if the calf took more than 1 min to start drinking from the bottle, the calf was gently guided toward the teat bottle. On the following training days, the calf was given 2 min to start drinking from the bottle; failure to approach was recorded as a non-approach. After each training session, calves were given the remaining milk of their morning ration in the home pen. Due to technical issues, 4 calves had a break between 2 training days and thus were provided one additional daily training session before the test phase.

#### Testing.

Following the 3 training days, sensitivity to reward loss was assessed. Test sessions were performed between 8:00 h and 9:00 h for 5 consecutive days using a procedure similar to the training sessions. Test sessions started with the calf held in the start box for 1 min and then the door was opened. The calf was then free to enter the arena and to approach the bottle but the ration was reduced to 0.1 L of milk. The test trial ended when the calf had consumed all the milk. A total of 3 consecutive trials were performed in each test session (providing a total of 0.3 L of whole milk in contrast with 1.5 L of milk available during the training sessions). After each test session, calves were given the remaining milk of their morning ration in the home pen.

During both training and test days, calves’ latencies to start drinking from the milk bottle were continuously recorded during each trial using a video camera (WV-CW504SP, Panasonic, Osaka, Japan) positioned at the center point 7 m above the arena. Latencies were recorded from videos by 2 observers blind to treatment. Inter-observer reliability was evaluated using the intra-class-correlation coefficient (ICC, package *ICC*); *ICC* was 0.997 based upon the assessment of a random selection of 35 videos assessed by both observers.

### Statistical analyses

All statistical analyses were conducted with *R*, version 4.3.1. All data and code are available (https://doi.org/10.5683/SP3/TKH4WT).

#### Social discrimination task.

During test sessions we recorded approaches as correct (approaching S+), or incorrect (approaching S-); values are reported per day and per calf. Our learning criterion was that calves approached correctly more often than not (i.e., > 5/10 correct approaches) over 4 consecutive sessions, the probability of such performance occurring being *P = *0.02 (Binomial distribution, probability parameter of 0.5). A linear mixed-effects model (*R* package *lme4*; *R* package *lmerTest*) was used to test the effect of treatment and test session (as fixed factors), including calf as random factor, on the number of correct answers. Residuals were visually assessed for normality using residual fitted plots and quantile-quantile plots.

**Fig 3:**
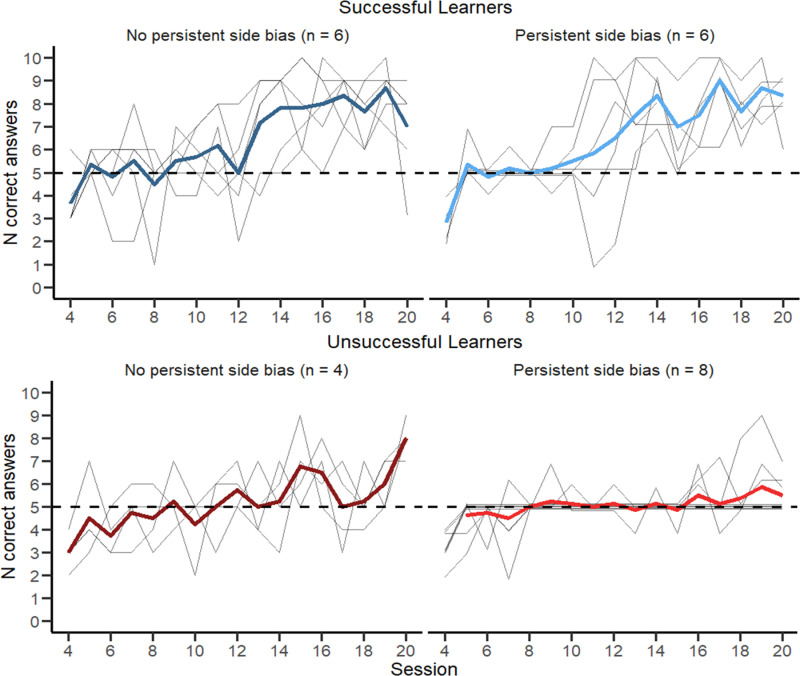
Number of correct answers in relation to test sessions for the discrimination task. Calves were classified successful learners if they reached the learning criterion, and as side biased if they approached only one side (i.e., left or right; regardless of the stimulus) for 2 or more sessions. Grey lines represent individual calves and colored lines the data averaged per group.

To capture the variation in learning curves among calves, we classified individuals according to their learning success (i.e., if they reached the learning criterion), and any persistent side bias (i.e., calves approaching only one side regardless of the stimulus for more than 2 sessions). Data presented in Fig 3 were averaged by category, i.e., successful learners with persistent side bias, successful learners without persistent side bias, unsuccessful learners with persistent side bias, and unsuccessful learners without persistent side bias.

#### Successive negative contrast.

Data (i.e., latency to start drinking per trial and per calf) from the 3 following phases of the Successive Negative Contrast test were included in the analysis: training (i.e., 3^rd^ training day), reward downshift: day 4 (i.e., 1^st^ day of reduced reward), and reward downshift: days 5–8. All calves drank milk during each trial. For each phase, residuals were checked for normality. A linear mixed-effect model was performed by phase (i.e., one model for each of the 3 phases) on latencies to reach the reward including treatment and daily trial (i.e., the order of trials performed within a day) as fixed factors, and calf as random factor. Post-hoc comparisons were realized on the training data: models included daily trial as fixed factors and calf as a random factor were performed on the data subgrouped by treatment. For the model including data from d5 to 8, latencies were log-transformed to fit model assumptions of residuals normality and test session was added as a fixed factor.

## Results

### Social discrimination task

**Fig 4:**
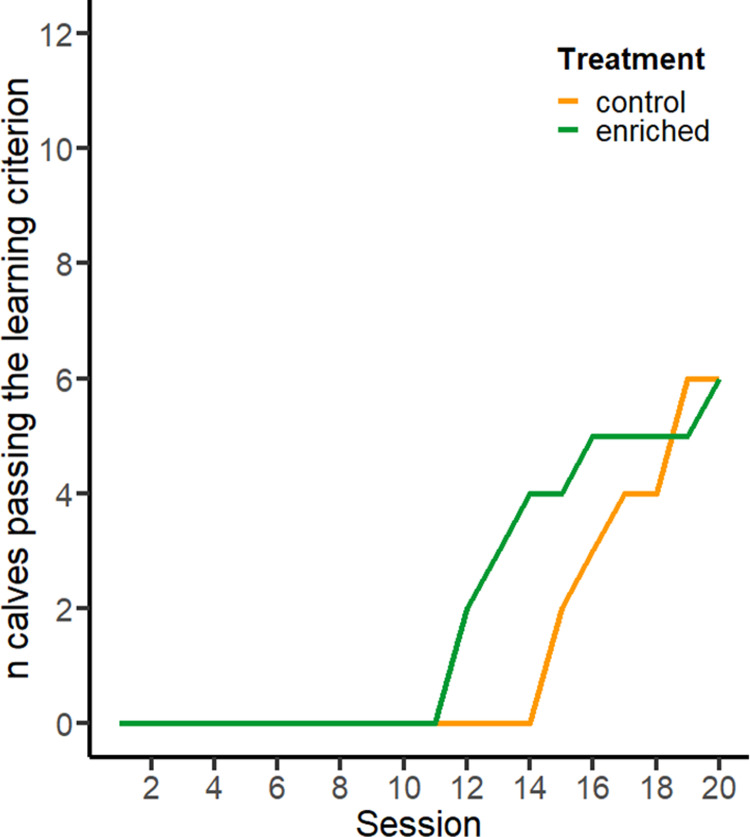
Number of calves that met the learning criterion, in relation to test sessions, for the social discrimination task. The discrimination task was considered learned when calves achieved > 5/10 correct responses during 4 consecutive sessions.

Twelve calves reached the learning criterion, requiring 15.7 ± 2.59 sessions (ranging from 12 to 20; Fig 4) to discriminate among calves. Learning success did not differ between treatments, as measured by pass rate (control: 6/12, enriched: 6/12) and the number of sessions required to meet the learning criterion (control: range 15–19, enriched: 12–20). Number of correct responses increased over test session (F_1,383 _= 219.29, *P* < 0.001) but there was no difference between treatments (F_1,10 _= 0.343, *P *= 0.558). Individual learning curves varied, with 20 calves showing evidence of a side bias over the course of the learning process, (i.e., approaching only the left or right side during an entire session, regardless of stimulus location). For 14 calves, side bias persisted for more than 2 sessions (i.e., persistent side bias), but this bias did not affect the learning success (6/12 successful learners and 8/12 unsuccessful learners showed persistent side bias; Fig 3). For 12 of the 20 calves that showed a side bias, the preferred side was the one where they were first rewarded in during the first test session.

### Successive negative contrast

**Fig 5:**
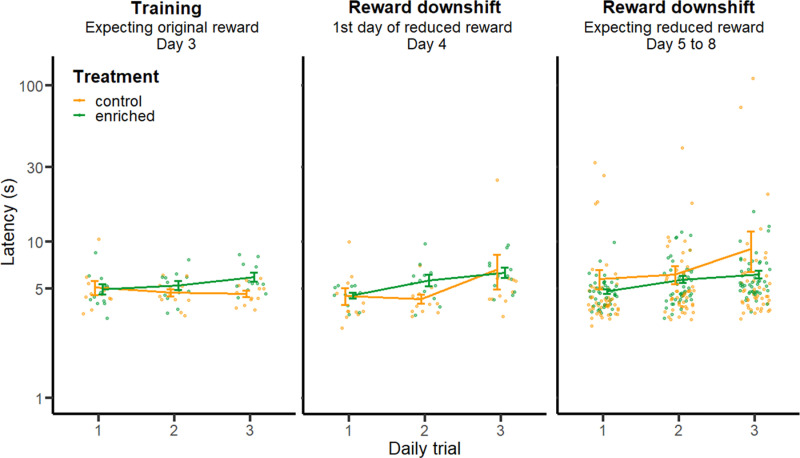
Calf latency to approach the milk reward over 3 daily trials, before and after a reward downshift. Bars illustrate the mean ± SE latency per treatment group and dots represent individuals’ data points. During training (day 1 to 3), calves received a reward of 0.5 L of milk. Starting on day 4, calves were given a reward reduced to 0.1L. From days 5 to 8, as they already had experienced the reduced reward, calves may have expected a reduced reward.

During the last training day (i.e., day 3), calf latency to approach the milk reward was affected by an interaction between treatment and daily trials (LMM with Anova, F_1,68_ = 7,87, *P *= 0.005; Fig 5). This interaction was driven by enriched calves increasing latencies over daily trials (F_1,34_ = 14.20, P < 0.001, slope = 0.48, SE = 0.19) whereas control calves did not (F_1,34_ = 1.05, *P *= 0.304, slope = 0.22, SE = 0.22). On test days, i.e., when the milk reward was reduced, calves increased latencies across trials (day 4: F_1,68_ = 18.60, *P *= 0.036, enriched slope = 0.90, SE = 0.16; control slope = 1.06, SE = 0.70; days 5–8: F_1,261_ = *P *< 0.001, enriched slope: 0.68, SE = 0.15; control slope = a: 1.62, SE = 1.05) but no effect of treatment (d4: F_1,68_ = 1.99, *P *= 0.158; d5 to 8: F_1,261_ = 0.03, *P* = 0.864) or session (d5 to 8: F_1,261_ = 1.04, *P *= 0.307) was noted.

## Discussion

### Discrimination task

To our knowledge, this is the first study to investigate social discrimination in calves. Twelve calves learned the discrimination task, suggesting that calves are able to distinguish individuals despite similarities in age, sex and breed. These findings align with studies showing adult dairy cows’ ability to discriminate among conspecifics [32,33]. Individual discrimination is also implied by calves being able to develop preferential relationships with peers [47,48]. We conclude that calves are capable of individual discrimination, a prerequisite for individual recognition [49,50]. We found no evidence that temporary access to a well-resourced pen affected social discrimination. Given the limited number of calves reaching the learning criterion, our study may have lacked power to detect this effect. Future work should take into account that likely not all animals will learn, and should estimate power using the number of animals that successfully learn the task. Previous work on dietary enrichment found improved calf performance in a spatial discrimination task during both initial learning [51] and reversal learning [18]. Environmental complexity is generally found to influence behavioral flexibility [52], often assessed via reversal learning. Gaillard et al. [17] reported impaired performance in reversal learning of a discrimination task for calves raised individually in comparison to pair-housed calves but did not find such a difference during the initial learning phase. Meagher et al. [16] compared individually reared calves with those kept with their dams in groups, and found no difference in the initial learning of a discrimination task but did observe improved performance in reversal learning by dam-reared calves.

Twelve of the 24 calves did not meet the learning criterion. We used unfamiliar live calves as stimuli to allow the use of cues from several sensory modalities to perform the task. Even though tested calves were repeatedly exposed to the stimuli, the experimental setup may have restricted the calves’ ability to perceive some of the stimuli characteristics. Access to partial cues may have increased the complexity of the discrimination task, resulting in some calves not learning in the time allowed during this study. Previous work has suggested that physical contact is necessary for calves to develop social bonds; visual [53], olfactory, and auditory cues alone [47] are not sufficient for the formation of social preferences. In our study, focal calves had only limited access to the stimulus calves during testing, which might not have been sufficient to develop familiarity. Familiarity can affect learning speed in recognition tasks; Coulon et al. [34] reported that heifers recognize familiar individuals more easily than unfamiliar ones. A similar effect has been described in sheep [54]. Learning the task also may have been impaired by the lack of ecological relevance; calves were trained to associate another calf with a milk reward when in their daily life calves often compete with each other for milk.

Many calves showed a side bias (i.e., repeatedly entering only one arm of the Y-maze during a test session) that persisted over sessions, but side bias was not associated with learning success. Animals often attempt to solve tasks using spatial cues [55], and cattle are known to develop position preferences [56]. Hosoi et al. [57] explored foraging strategies in a Y-maze and reported that most cows spontaneously used a win-stay strategy (i.e., returning to the same arm in which they received a reward) if all arms were rewarded by food before modifying their foraging behaviors to a win-shift strategy (i.e., after being rewarded in one arm, entering the other arm) in response to the possibility of losing resources. Lecorps et al. [58] argued that hunger affects a calf’s ability to focus, and found that hungry calves tested in a spatial task revisited unrewarded locations more often than did satiated calves. In the current study calves had not been fed their morning milk-meal before testing, potentially interfering with the task.

### Successive negative contrast

The shorter latencies of control calves to approach the milk bottle (before the reward downshift) is consistent with a greater motivation to access the reward, an indicator of negative emotional state [59]. One previous study on cattle [60] found that indoor-housed cows approached reward buckets more rapidly than did cows that had access to pasture. Neave et al. [42] found that calves reared in basic pens approached a reward pen more rapidly, and expressed more anticipatory behavior before access, than did calves kept in enriched housing. Rats reared in standard cages showed more anticipation before accessing a reward than did rats housed in semi-naturalistic environments [61]. Together these findings indicate that more barren housing is associated with increased sensitivity to reward, in a manner consistent with these animals experiencing more negative emotional states.

In the current study, the experience of being in the test arena itself may have been more valuable for control calves than the enriched ones, given that this was their sole opportunity to run. All calves were housed in pens too small to allow locomotory play [62], but the enriched calves could perform those behaviors once a day when accessing the well-resourced pen, while control calves did not. Many studies have described the effect of home pen size on locomotory play when calves are released in a larger test arena [21,22,62]. In another study, Sutherland et al. [63] compared running durations in a test arena between calves reared socially but with different space allowances: calves housed in smaller pens ran longer during the test than did calves reared in larger pens. Rushen & de Passillé [64] also describe a rebound in locomotory play in an arena when calves experience a spatial restriction in the home pen.

Once the milk reward was reduced both control and enriched calves increased latencies over repeated trials within each test day. Following other studies using Successive Negative Contrast tests on various species (rats: [40]; pigs: [41]; cattle: [46], such results are expected and suggest that animals experienced a frustration-like response to reward loss. Over the entire reward downshift phase, calves’ latencies did not differ among treatments, contrasting with previous work investigating the effect of the environment on sensitivity to reward loss. For example, Burman et al. [40] found that conventionally housed rats express a prolonged response after a reward downshift, characterized by increased latencies to approach the reward over several days when enriched rats showed a rebound in speed only after two days of reduced reward. Pigs raised in barren housing early in life responded more slowly after a reward downshift but no such effect was reported for pigs reared in enriched environments [41]. Both of these studies used a longer training period than used in our study. The more limited training period may have prevented calves from developing reward expectations. In addition, our relatively small arena may have failed to capture speed variation among individuals.

Enriched calves increased latencies over daily trials before and after the loss of reward. In contrast, control calves changed their response pattern after the reduction of reward: they showed consistent latencies over daily trials during the initial reward phase but became slower when the reward was reduced. We speculate that this pattern suggests a stronger response to the loss of reward from control calves than enriched calves.

Previous work supports the negative effect of a poor environment on animals’ affective states. In rats, providing enrichment items [40], or semi-naturalistic environments [61] has been shown to promote positive affective states and to be highly rewarding [44]. Consistent results have been reported in pigs with physical enrichments inducing optimistic judgment biases [26] and reduced sensitivity to reward loss [41]. Calves appear to develop a pessimistic bias when reared individually [25] and increased sensitivity to reward when housed in restricting environments [42]. Work investigating whether pasture access impacts judgment bias in dairy cows [60] reported contrasting results. Despite an increased reward sensitivity for cows housed indoors compared to individuals that had access to pasture (i.e., a more complex environment), judgment bias did not differ among treatments. In our study, the null results following the reward loss could also be explained by treatments not diverging enough to induce strong differences in mood states. All calves had experienced 20 days of training for the discrimination task prior to being tested for Successive Negative Contrast. Considering that cattle have been shown to experience learning tasks as rewarding [65], the discrimination task likely contributed to enriching calves’ early-life experience. All calves experienced this training, perhaps mitigating the effect of treatment on affective states. Additionally, we encourage future work to include other behavioral measures such as the number of vocalizations and pressure applied to the milk bottle [46]; including these measures may have increased our ability to identify differences in calves’ responses to reward loss.

## Conclusions

Calves were able to discriminate among individuals, but contrary to our prediction, temporary access to a well-resourced environment did not improve their ability to learn this task. Calves provided access to the well-resourced pen showed reduced sensitivity to reward compared to control calves, but no difference was found in sensitivity to reward loss. Our findings suggest that even temporary access to a well-resourced environment can affect dairy calves’ affective state.

## Supporting information

S1 Fig Timeline of the discrimination task and successive negative contrast tests. (TIF)
