# Supplementary figures and images for: Effects of access to a well-resourced environment on dairy calf cognition and affective state
Source: PLoS One. 2025 May 16;20(5):e0323089. doi: 10.1371/journal.pone.0323089 (PMC12083789; doi:10.1371/journal.pone.0323089)

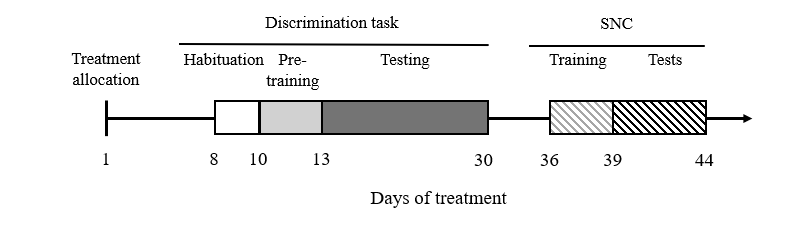

Supplement: S1 Fig — (TIF) [file pone.0323089.s001.tif]
